# Polypus Uteri

**Published:** 1844-06-01

**Authors:** 


POLYPUS UTERI.

M. Lisfranc makes the following remarks on this disease:—When polypus uteri is complicated with anemia from repeated hæmorrhage, and when the least loss of blood may occasion fatal consequences, it is impossible to use the knife for its removal, on account of the hæmorrhage which may ensue; or the ligature, because, it may cut the pedicle like a knife, or may include a portion of the uterus when the pedicle is formed wholly or partly of the tissue of that organ; or it may be applied, but with considerable difficulty, and produce serious accidents; and, finally, it may give rise to injurious effects from the presence of the instruments in the vagina, and the fœtid matter resulting from the sphacelus of the tumour. I would advise the following treatment, varying it according to the seat of the polypus.

*Polypus situated in the vagina.*—If the strength is not too reduced, the tumour may be removed, but if hæmorrhage exists and the size of the polypus be such as to permit its being reached, it ought to be cauterized with the protonitrate of mercury so as to stop the flow of blood; this attained, the patient gradually gains strength, after which the customary operation may be performed with infinitely less danger. If, on the contrary, the tumour is so large as to render it impossible to cauterize the whole surface, the ligature must be employed.

*Polypi contained partly or entirely in the uterus.*—In the former, after introducing a speculum, the part contained in the vagina must be cauterized; this I have always found sufficient to stop the hæmorrhage. In the latter, the cervix uteri must first be dilated and the proto-nitrate employed; the blood ceases to flow almost immediately, though but a very small portion of the polypus be cauterized: this is easily explained, it being pressed on all sides by the uterus, and the blood can only proceed from the part corresponding to the cervix uteri. When the cauterization checks the hæmorrhage, the polypus ought not to be removed before the strength of the patient be such as to preclude all danger; should it however not suffice, the ligature must be applied immediately. It sometimes happens when the polypus is entirely in the uterus, that the hæmorrhage continues to such an extent as to put the life of the patient in danger, and that it is impossible to dilate the cervix. Here death may ensue if relief is not procured; consequently, the cervix uteri must be seized with a sharp hook (erigne,) drawn gradually and gently down to the labia pudendi and divided, after which the ligature, knife, &c., may be employed according to circumstances. In these last, the danger is greater and relapses more frequent than when the tumour is in the vagina.—*Ibid.*

